# Perspectives of Challenges in Counseling for Congenital Heart Defects

**DOI:** 10.1007/s00246-024-03520-x

**Published:** 2024-06-22

**Authors:** Joyce L. Woo, Rupali Gandhi, Shelvonne Burton, Adithya Sivakumar, Sarah Spiewak, Renee Wakulski, William A. Grobman, Matthew M. Davis, Angira Patel, Joyce T. Johnson, Stefani Samples, Lynn M. Yee

**Affiliations:** 1 Division of Cardiology, Department of Pediatrics, Ann & Robert H. Lurie Children’s Hospital of Chicago,, Northwestern University Feinberg School of Medicine, 225 E. Chicago Ave, Box 21, Chicago, IL 60611 USA; 2 Department of Medical Social Sciences, Northwestern University Feinberg School of Medicine, Chicago, IL USA; 3 Division of Cardiology, Department of Pediatrics, Advocate Christ Children’s Hospital, Chicago, IL USA; 4 Section of Cardiology, Department of Pediatrics, University of Chicago, Chicago, IL USA; 5 University of Maryland Baltimore County, Baltimore, MD USA; 6 Rush Medical College, Chicago, IL USA; 7 Department of Pediatrics, Advocate Christ Children’s Hospital, Chicago, IL USA; 8 Division of Maternal-Fetal-Medicine, Department of Obstetrics and Gynecology, The Ohio State University College of Medicine, Columbus, OH USA; 9 Nemours Children’s Health, Wilmington, DE USA; 10 Division of Cardiology, Department of Pediatrics, Johns Hopkins All Children’s Hospital, St. Petersburg, FL USA; 11 Division of Maternal-Fetal-Medicine, Department of Obstetrics and Gynecology, Northwestern University Feinberg School of Medicine, Chicago, IL USA

**Keywords:** Counseling, Prenatal diagnosis, Congenital heart defects

## Abstract

Prior work regarding counseling patients about congenital heart defects (CHD) has focused on their perceptions about accurate communication of cardiac anatomy, and the emotional support received from the provider. The objectives of this study were to identify the additional CHD counseling-specific challenges and areas for future intervention, using a practical communication framework. This is a secondary analysis of qualitative data provided by caretakers of infants who received congenital heart surgery from 2019 to 2020 in the Chicagoland area. While the survey in the primary study pertained to barriers in obtaining prenatal diagnosis, respondents with *both* prenatal and postnatal diagnosis reported challenges to effective counseling. Qualitative data measuring counseling challenges were collected from semi-structured phone interviews. Thematic analysis was performed using an inductive approach. Themes were organized into five domains using SPIKES (Setting, Perception, Invitation, Knowledge, Empathy, and Summarize/Strategy), a previously validated framework to help clinicians effectively break bad news. Among 160 survey respondents, 35 (21.9%) reported a challenge during CHD counseling that they received. In total, 12 challenges were identified and spanned all six SPIKES domains. The three most common challenges were as follows: perception of repeated imaging studies for accurate diagnosis or management (*n* = 19, Knowledge), the lack of cardiologist presence at the time of initial CHD detection (*n* = 8, Setting), and insufficient information provided about the CHD diagnosis (*n* = 7, Knowledge). Patients perceive counseling as a key component of prenatal diagnosis of CHD and identify the challenges that exist at all stages of the counseling process. These findings suggest that effective counseling extends beyond conveying information about anatomy and prognosis.

**Supplementary Information:**

The online version contains supplementary material available at 10.1007/s00246-024-03520-x.

## Introduction

Congenital heart defects (CHDs) are the most common and resource-intensive birth defects in the United States [1, 2]. Diagnosis and management of CHD require two phases of care: accurate identification of the CHD followed by effective counseling about the diagnosis [3, 4]. Counseling includes process components, such as delivering the unexpected information (e.g., having the correct clinician present, acknowledging emotions, addressing questions), and content components, such as explanations of the anatomy, options for management (e.g., palliative care, surgery), short-term outcomes (e.g., potential surgical complications), long-term outcomes (e.g., life expectancy, neurodevelopmental outcomes), and genetic associations. For prenatal counseling, fetal monitoring during the pregnancy and decisions about termination and delivery are additional topics that fetal cardiologists must address [5].

Compared to research in the accurate identification of a CHD, research in effective counseling for CHD is nascent [5, 6]. Most research pertaining to counseling addresses either the patient’s understanding of cardiac anatomy as a proxy measurement of the provider’s communication skills [4, 7–9], or the patient’s perception of emotional support from the provider following a new diagnosis of CHD [10, 11]. Yet effective counseling extends beyond these factors, and parents may weigh the importance of counseling topics differently than providers [12]. To improve and standardize counseling practices, more knowledge of patient perceptions of challenges in CHD counseling is necessary.

Our research team previously investigated patient-reported socioeconomic barriers to prenatal identification of a CHD using interviews [13]. The objective of this analysis was to describe the patient-reported challenges to effective prenatal *or* postnatal counseling that also emerged during these interviews, using a previously validated, practical communications framework.

## Methods

### Study Design and Inclusion Criteria

This is a secondary, qualitative analysis of data generated during a study that evaluated patient-reported barriers to prenatal identification of CHD [13]. Study participants were caretakers of infants who received their first congenital heart surgery requiring cardiopulmonary bypass between 0 and 12 months of life, at Advocate Christ Children’s Hospital (Advocate) or Ann & Robert H. Lurie Children’s Hospital of Chicago (Lurie Children’s) from January 1, 2019 to December 31, 2020. Caretakers of infants born on or after March 11, 2020, the start of the COVID-19 pandemic, were included. Caretakers of infants whose surgeries did not require cardiopulmonary bypass (e.g., ligation of patent ductus arteriosus) were excluded. Caretakers with an inactive phone number, who preferred to complete the survey in American Sign Language, or who no longer had primary custody of the infant, were excluded.

Advocate and Lurie Children’s capture a demographically and geographically diverse patient population within the Chicago Metropolitan Statistical Area (MSA). This is the third-largest MSA in the United States by population, and the tenth most diverse MSA by race and ethnicity based on 2010 US Census data [14]. Additionally, Advocate and Lurie Children’s serve patients from rural Illinois, Iowa, Wisconsin, and Northwest Indiana.

### Survey Data and Collection

A summary of the survey data utilized for this analysis has been previously described [13]. In brief, a semi-structured survey was created with an evidence-based design [15] and input from qualitative researchers, pediatric/fetal cardiologists, and a maternal–fetal medicine specialist (RG, JTJ, AP, JLW, LMY). The survey contained 13 closed-ended questions and one open-ended question pertaining to prenatal care and barriers to prenatal diagnosis of CHD (Supplemental Table 1). In the open-ended question, respondents were asked to respond in their own words regarding their experience of prenatal CHD diagnosis (“Is there anything else you’d like to mention about how congenital heart disease was diagnosed during your pregnancy?”). Following their response, data collection specialists (SB, AS, SS, RW) were trained to ask follow-up questions (e.g., “What do you mean when you say that that you were unhappy with the information provided?”). These specialists were trained to encourage information about counseling from all types of specialties (e.g., cardiologists, neonatologists, nurses, obstetricians). These answers were then entered with free text into the data collection form. Subsequent thematic analysis pertains only to responses to this open-ended question.

All surveys were administered via phone between May 2022 and February 2023. The mean time between the infant’s date of birth and the date of survey was 2.9 years (SD 0.6 years). As caretakers of infants who died or experienced major complications were included, we chose to begin recruitment in May 2022 to allow for at least 2 years of potential bereavement. Attempts to contact each caretaker by phone were made at least twice. Phone interpreters were employed for respondents who preferred to complete the survey in a language other than English. All survey data were entered into Research Electronic Data Capture [16] (REDcap, Northwestern University Clinical and Translational Sciences Institute). Data collection commenced in May 2022 and ended in February 2023.

### Thematic Analysis

The thematic analysis focused on organizing and describing the challenges to counseling and prenatal management. Responses to the open-ended question were first independently codified into excerpts by a team of primary coders (SB, AS, JW). Disagreement in code was discussed among the primary coders until agreement was achieved. Initial thematic analysis was conducted by the primary thematic analysis team (JW and RG), who re-evaluated all excerpts to confirm consistency by reading the entire dataset multiple times, then organized the excerpts into themes. Example themes include “Insufficient information provided about CHD diagnosis” or “Unknown specialty of the counseling provider.” Each respondent could contribute multiple excerpts, but each excerpt could only contribute to one theme. A theme was defined by two or more respondents each contributing an excerpt related to one concept. Concepts represented by a single respondent were reported if considered particularly salient, despite not meeting criteria for a theme.

A secondary thematic analysis team (JW and LMY) then organized each theme into one or more domains following the SPIKES framework, with specific consideration given to the context of CHD counseling. Definitions of each domain of the SPIKES framework, with an example excerpt, can be found in Table 1. The SPIKES framework was originally developed by Baile et al. as a protocol to assist oncologists in the delivery of bad news to patients with an initial cancer diagnosis, and has since been demonstrated to improve provider performance in counseling and patient knowledge of non-oncologic diagnoses also [17, 18]. SPIKES is considered both a practical communication framework with direct applicability to clinical care, and a theoretical framework to organize data regarding communication and counseling. After organizing themes by SPIKES domain, reflexivity was addressed with evaluation of all domains and their corresponding themes by the tertiary thematic analyst team, which included obstetric (WAG), cardiology (JTJ, SS), and qualitative analysis (WAG, MMD) expertise. Each theme could contribute to multiple SPIKES domains.

**Table 1:** Domains of the SPIKES communication framework, with example excerpts

Domain	Definition	Example excerpts of a challenge in counseling
Setting	Setting up the interview by choosing an appropriate environment, timing, and individuals important to the patient to have a conversation with the patient	Parents were unable to meet with the pediatric cardiologist after fetal echo due to [the cardiologist’s] schedule, it took hours for the doctor to come and both of parents needed to get back to work
		Diagnosed with postnatal echo. No pediatric cardiologist [at the community hospital where infant was born], and the information relayed was very vague
Perception	Using open-ended questions to assess the patient’s perception including knowledge, expectations, and hopes	Even with multiple ultrasounds, no one explained it was because the baby’s heart views were incomplete. They kept saying everything was fine
		Constant trips for echocardiograms were the most difficult part, and they didn’t change anything
Invitation	Assessing how much information the patient would like to receive, and to what level of detail	Patient told they needed follow-up within a specific time period as it was the cutoff for termination. However, patient felt that this was an inappropriate comment as they were clear that they would not terminate their pregnancy
		Abortion was not an option for them… she felt unsupported and felt like she had to fight for their child to get good medical care
Knowledge	Providing information about the diagnosis, management, and prognosis in plain language	The neonatologist told the family to Google Truncus before care was transferred to another hospital
		She thought she could have been more prepared for left vocal cord nerve paralysis and need for feeding tube after surgery
Empathy	Exploring and addressing the patient’s emotions with an empathic response	The MFM said she was making it [concerns for congenital heart disease on level 1 ultrasound] up, and was being too overwhelmed. Then they did the 20-week ultrasound and found a heart defect
		Patient did not see postnatal diagnosis as a negative, said it would have only stressed them out
Summarize/strategy	Checking the patient’s understanding of the information and explaining next steps	They found a heart defect at 26 weeks, had to see MFM a lot during pregnancy yet still wasn’t told the right place to deliver until the third trimester
		At the 20-week ultrasound, they saw something indicative of Down syndrome. It took an amniocentesis and so many imaging studies in order to actually diagnose the [heart] disease

To protect patient privacy, specific CHD diagnoses are reported within the text in a more generalized manner (e.g., tetralogy of Fallot with pulmonary stenosis may be reported as a conotruncal defect or non-critical CHD) unless four or more respondents had a fetus with the same diagnosis. The overall sample size for this analysis was constrained by the sample for the primary study, although thematic saturation was felt to have occurred within this sample. This study was approved by the Institutional Review Boards of Advocate Christ Children’s Hospital (1714144-5) and Ann & Robert H. Lurie Children’s Hospital of Chicago (2021-4303).

## Results

### Cohort Characteristics

In total, 160 individuals responded to the survey. The sociodemographic and clinical characteristics of survey respondents and non-respondents have been previously described [13]. Among survey respondents, 35 (21.9%) reported a challenge in the counseling they received for CHD diagnosis and/or management. The majority of this group who perceived a challenge in their counseling had a prenatal diagnosis (*n* = 28) came from an English-speaking household (*n* = 25), were of non-Hispanic White race and ethnicity (*n* = 22), and had an infant with a concomitant noncardiac abnormality, chromosomal abnormality, or genetic syndrome (*n* = 23, Table 2). All respondents who reported a counseling challenge were the birthing parents.

**Table 2:** Cohort characteristics of patients reporting challenge(s) in counseling or prenatal management of CHD (*N* = 35)

	*n* (%) or mean (± SD)
Prenatal diagnosis received	28 (80.0)
Maternal age, years	32.7 (± .0)
Maternal years of education	14.9 (3.0)
Maternal race/ethnicity	
Non-Hispanic White	22 (62.9)
Hispanic	6 (17.1)
Non-White	7 (20.0)
Household language	
English	25 (71.4)
Spanish	3 (8.6)
Other*	7 (20.0)
Nulliparous	13 (37)
Infant noncardiac abnormality, chromosomal abnormality, or syndrome	23 (65.7)
Congenital heart defect	
Non-critical	18 (51.4)
Critical or ductal-dependent^a^	17 (48.6)

*Other languages included Gujarati, Odia, Polish, Russian, and Ukrainian

^a^Critical CHDs include D-transposition of the great arteries, truncus arteriosus, hypoplastic left heart syndrome, total anomalous pulmonary venous return, and Ebstein anomaly

### Setting, Perception, and Invitation

**Fig. 1:**
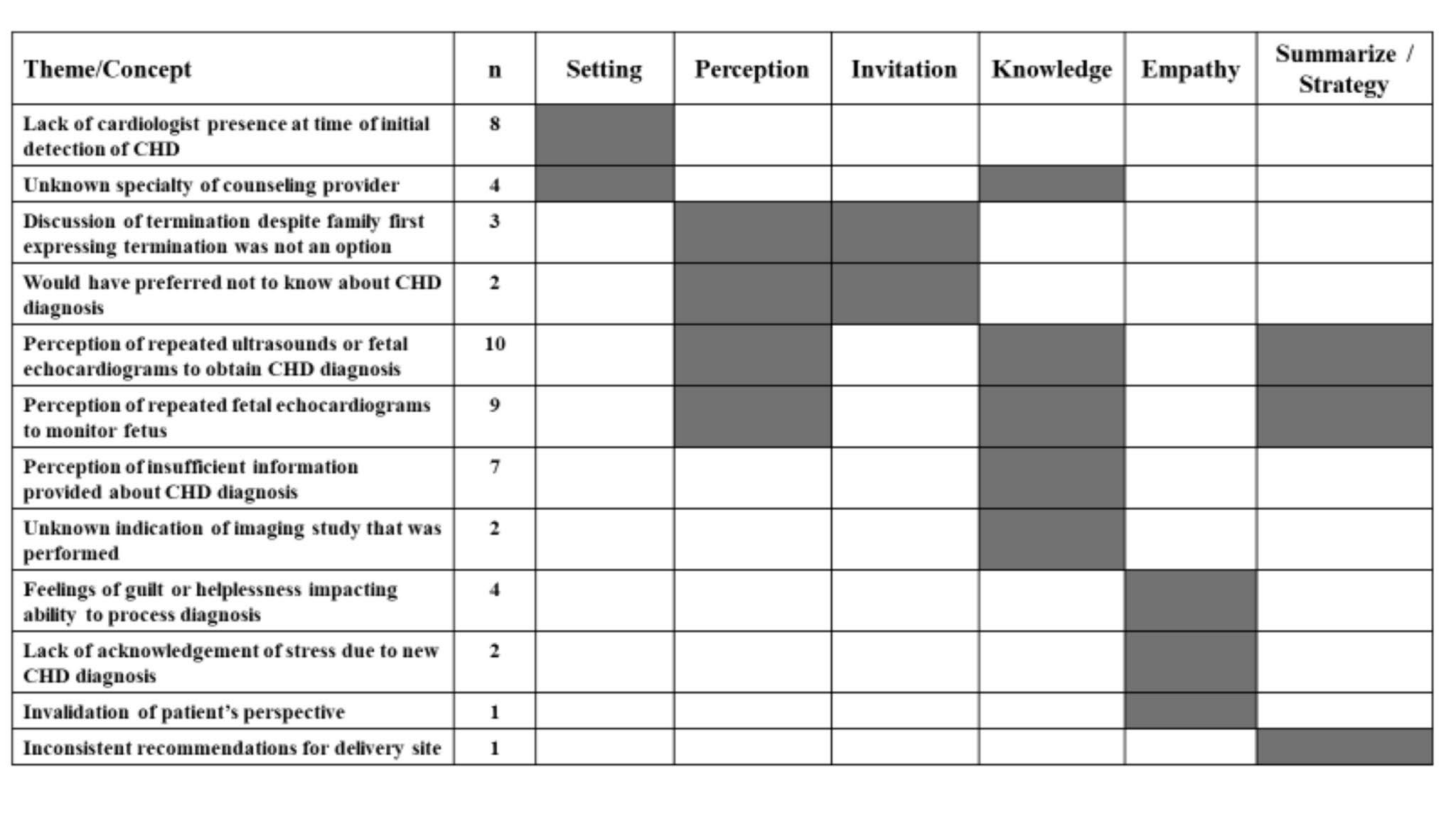
Patient-reported challenges to effective CHD counseling, categorized by the SPIKES framework

Next, we describe the themes/challenges in counseling and how they map to the domains of the SPIKES framework (Fig. 1). Two challenges emerged that related to the appropriate setting for counseling. First was the lack of cardiologist presence at the time when CHD was initially detected (*n* = 8). Six respondents reported that they wished a cardiologist had been present during their initial prenatal or postnatal counseling with an obstetrician or neonatologist, respectively. One respondent reported that a cardiologist was not available for counseling for several days following their fetal echocardiogram. One respondent had an infant with a postnatal diagnosis of CHD and did not receive any counseling from a cardiologist until the time of consent for their infant’s surgery, which included both repair of the CHD and the infant’s other, more significant, noncardiac congenital anomaly. This circumstance highlighted the challenges of counseling when multiple anomalies are present.

The second challenge was the lack of knowledge about the specialty of the provider who delivered their prenatal CHD counseling (*n* = 4). Two respondents were unsure if they ever received a fetal echocardiogram in addition to their second-trimester obstetric ultrasound, though a fetal echocardiogram report was found in their electronic health record. This challenge was dually categorized under knowledge, as it also related to patient knowledge of the expertise of those providing CHD counseling.

Two themes were related to the patients’ expectations (perception) and desire for information (invitation) during counseling. First, three respondents reported that their providers continued to discuss termination even though the respondents recalled they had already communicated that this was not an option for them. For example, one respondent reported that she was told that “she needed a follow-up within a specific time period as it was the cutoff for termination. However, [she] felt that this was an inappropriate comment as [she] was clear that she would not terminate her pregnancy.” Second, two respondents reported that they would have preferred not knowing, or were satisfied without knowing, about their fetus’ CHD prior to delivery. Both respondents had fetuses with non-critical CHD that is typically repaired around 6 months of age; one respondent had a prenatal diagnosis while the other had a postnatal diagnosis.

### Knowledge

Five challenges emerged in communicating knowledge about the CHD diagnosis and its management. The most frequent was the perception of repeated imaging studies to either obtain the correct prenatal diagnosis (*n* = 10) or monitor the fetus (*n* = 9). Both challenges were dually categorized under the domains of perception and summarize/strategy because they also related to patient expectations for subsequent imaging (perception) and could potentially represent communication gaps in conveying the indications for subsequent imaging for prenatal management (summarize/strategy, Fig. 1). Among the 10 respondents who reported repeated imaging to obtain the correct CHD diagnosis, four specifically noted repeated fetal echocardiograms: all of these respondents had fetuses with tetralogy of Fallot with pulmonary stenosis or tetralogy-type double outlet right ventricle. Because there is little physiologic significance to precise anatomic delineation of these two diagnoses prenatally, it is likely that the cardiologists’ recommendations for follow-up fetal echocardiograms were to evaluate the degree of right-sided obstruction. Respondents were not asked follow-up questions regarding why they perceived the fetal echocardiograms as repetitive.

Among the nine respondents who perceived that they had repeated fetal echocardiograms for monitoring, three had fetuses whose systemic circulations were dependent on an unrestrictive atrial septum, and three had fetuses with evolving outflow obstruction. The documented clinical need for follow-up echocardiograms for these diagnoses suggested the presence of communication gaps regarding the indication for follow-up fetal echocardiograms. In contrast, the remaining three had fetuses with isolated septal defects, which are less likely to impact cardiopulmonary physiology in the neonatal period.

The third most common challenge pertaining to knowledge was that patients perceived that their counseling provider (*n* = 7) provided insufficient or inconsistent information. For example, one respondent recalled that they were initially told by their obstetrician that their fetus’ CHD was “not concerning,” but ultimately had a diagnosis of a critical CHD. Another respondent felt that the obstetrician’s explanation of CHD was insufficient and was surprised when the fetal echocardiogram demonstrated a non-critical defect. One respondent wished that the pediatric cardiologist had warned her more prenatally about the possibility of vocal cord paralysis and placement of a nasogastric feeding tube following their child’s aortic arch repair. Two respondents had a postnatal diagnosis and were initially counseled by a neonatologist; one respondent who delivered at a center without a pediatric cardiologist available recalled being told to “Google” the critical diagnosis prior to the neonate’s transfer to a tertiary center.

### Empathy and Summarize/Strategy

Two themes emerged that pertained to stress associated with CHD counseling (Empathy): feelings of helplessness or guilt impacting the respondent’s ability to process the diagnosis (*n* = 4) and lack of acknowledgement of stress due to a new diagnosis of fetal CHD (*n* = 2, Fig. 1). Although these themes pertained to individual’s experience, it was included as a challenge because it reflects the individual’s ability to process information conveyed during counseling. Among those respondents who reported helplessness or guilt, one elaborated that they felt that “nothing could be done before the baby was born,” while another elaborated that they were concerned “it was my fault,” and even after successful postnatal surgery, still reported wondering “if I did anything to cause it [critical fetal CHD].”

Two respondents reported that their provider *teams* did not acknowledge the stress associated with CHD diagnosis. One respondent had a late prenatal diagnosis of critical CHD at 36 weeks, which required induction of labor at 37 weeks. They felt that the delivery team did not acknowledge the degree of stress due to a new CHD diagnosis. A second respondent had an infant with postnatal diagnosis of critical CHD requiring emergent intubation at a community hospital and transfer to a CHD surgical center. The respondent felt that the neonatology team at the delivery hospital did not acknowledge the stress and trauma associated with postnatal CHD diagnosis. Both respondents did not elaborate further regarding the specific actions and behaviors that contributed to their impressions.

Two concepts were not characterized as themes due to low representation in this sample but warrant discussion as they raise salient points about potential challenges in effective counseling: invalidation of the patient’s perspective (*n* = 1) and inconsistent recommendations for a delivery site (*n* = 1, Fig. 1). One respondent reported feeling invalidated when their obstetrician had counseled that CHD was present following their first ultrasound, but prior to their follow-up targeted ultrasound, was told by their maternal–fetal medicine specialist that “I was making it [the diagnosis of CHD] up.” One respondent reported that despite multiple visits with their maternal–fetal medicine specialist following diagnosis of fetal CHD, the appropriate delivery hospital wasn’t determined until the third trimester.

## Discussion

In response to an open-ended question pertaining to CHD diagnosis, nearly one-quarter reported a challenge related specifically to prenatal or postnatal counseling, reflecting patients’ value of effective counseling as *part of* CHD diagnosis. That challenges were reported from those with either prenatal or postnatal diagnosis signifies the need for improved counseling practices across many clinical subspecialities and phases of care. Finally, using the SPIKES framework [17], we demonstrate that patients value aspects of counseling that are not directly related to CHD anatomy or outcomes, such as more information about their counseling provider’s specialty (setting), and the counseling provider taking into account their expectations for information about the diagnosis or management decisions (perception and invitation). More specifically, we identified two areas for improvement that have not been previously described [6]: challenges in communicating the indications for studies, which may have led to the perception of repetitive and unnecessary imaging studies, and the lack of cardiologist input at the time of initial CHD diagnosis.

Significant research has been dedicated to increasing the accuracy of identifying congenital heart defects, including incorporation of outflow tract views on obstetric ultrasound to improve the detection of conotruncal defects [19, 20], and the use of artificial intelligence to detect CHD [21, 22]. By comparison, research pertaining to counseling expectant parents about CHDs is nascent [6]. Previous studies pertaining to counseling have quantitatively evaluated communication skills by measuring patients’ knowledge of the cardiac anatomy and prognosis, rather than other essential aspects of counseling [7, 9]. In this hypothesis-generating analysis, perception of repetitive studies might have been mitigated with more counseling pertaining to the indication for follow-up studies. Implementation research that tests these types of counseling interventions will better inform the development of formal curricula for CHD counseling, which does not currently exist for trainees, but has been proposed as an educational priority [5, 8].

The most common counseling challenges that we identified related to the effective communication of indications for imaging. Some respondents directly stated that they did not know the specialty of their counseling provider or the reason that an imaging study was performed. Others reported a perception of unnecessary repetitive imaging studies. Among those who reported repeated fetal echocardiograms to obtain the correct anatomic diagnosis, all had fetuses with either tetralogy of Fallot or tetralogy-type double outlet right ventricle, where the cardiologists’ reasoning for follow-up echocardiograms were likely to evaluate the degree of right-sided outflow obstruction, rather than to delineate anatomy. Among those who reported repeated imaging for monitoring, most had critical fetal CHD with potential for evolving outflow tract obstruction or restrictive atrial septum. While only some of the patients who reported repeated imaging for monitoring specifically stated the perception that the repeated imaging did not change management, clearly communicating the indication and importance of follow-up studies may have prevented these perceptions of repeated imaging and subsequently improved patient–provider relationships; inadequate communication of the indication could lead to frustration or even distrust, as suggested by these findings. Additionally, understanding testing indications might influence adherence to recommendations; in a previous analysis of this cohort, some respondents with postnatal or late CHD diagnosis reported that they forwent second-trimester obstetric ultrasound because it was normal for previous pregnancies, or they did not think it would add diagnostic value beyond routine blood tests obtained during pregnancy [13].

Conversely, a minority of respondents who reported repeated imaging had fetuses with isolated septal defects, which, in absence of other comorbidities, does not typically require multiple fetal echocardiograms to make decisions about perinatal management. More research is necessary to evaluate how repeated studies influence the pregnant individual’s perceptions about the CHD diagnosis, such as whether pregnant individuals perceive repeated studies as helpful, even if repeated studies were not necessary to make decisions about perinatal management. If there were some perceived benefit, an analysis of the increased prenatal expenditures due to repeated imaging against the value of the benefit for the pregnant individual (i.e., cost–benefit analysis) would also be highly informative.

The second-most common counseling challenge related to the lack of cardiologist presence at the time of CHD diagnosis, including the perception that insufficient information about the CHD was conveyed by the obstetrician, delivery team, or neonatologist. This finding is closely related to previous analyses that demonstrated that shorter length of time between ultrasound and counseling for CHD by a cardiologist [23] was associated with more patient-perceived success in counseling [24, 25]. Remote counseling from a cardiologist through telemedicine might mitigate both challenges. However, the feasibility of telemedicine for CHD counseling requires more understanding of the current supply/workforce of cardiologists, the demand for remote counseling, the cost–benefit of remote review of obstetric ultrasounds or echocardiograms performed at community hospitals [26], and the ethical and legal implications of remote counseling, such as misdiagnosis or the inability to fully address cultural norms or emotional cues in a remote setting [27]. Given these current limitations of telemedicine, improved training regarding patient-centered counseling from non-cardiologists may be one way to mitigate patient anxiety due to the lack of cardiologist presence.

Some challenges identified in our analysis were similar to those identified in previous studies, such as patients not feeling supported in their choices with regard to termination of pregnancy [28, 29]. This challenge further highlights the vital importance of strengthening provider training and comfort with communication about reproductive choice and patient autonomy, including for non-obstetric providers who co-manage CHD. This finding also highlights the strength of having multidisciplinary teams present when counseling pregnant individuals with a diagnosis of fetal CHD [30], a strength that was reported by several respondents in our study.

There were also findings from our analysis that differed from findings of previous studies, which may reflect study limitations. First, none of the respondents reported challenges related to language or health literacy in response to a broad question about barriers to diagnosis [31, 32]. These differences might reflect the impacts of resource investment for psychosocial support and translation services at our institutions that more pressing challenges overruled those related to language or health literacy, or selection bias within our respondent population. Second, approximately one-quarter of the respondents would have received prenatal care during the COVID-19 pandemic. While respondents previously described the lack of a partner as a barrier to diagnosis [13], specific counseling-related challenges during the COVID-19 pandemic were not identified in our analysis. Third, our analysis focused on challenges. A positive health approach focused on strengths and assets may warrant additional clinically useful findings. For example, three respondents remarked on the detail of the anatomic diagnosis and the accuracy of postnatal events that were explained previously. Another two respondents commented on the value of the multidisciplinary counseling for their fetuses, both of which had chromosomal abnormalities. However, due to the nature of our study design, further inquiry about these strengths was not conducted. Finally, we did not explore all potential counseling-related experiences, as our analysis includes responses to an open-ended question about barriers to CHD diagnosis. Future studies could utilize our findings and analytic approach using SPIKES, to design future work covering the full breadth of counseling experiences.

## Conclusion

Counseling is a key component of prenatal diagnosis of CHD, and our data demonstrate the value patients place on effective counseling. Although communicating knowledge about the anatomy and prognosis of CHD is important, other aspects of the counseling process and content, such as inclusion of cardiologist input at the time of initial diagnosis, and counseling about indications for imaging studies, are also key components for successful counseling from the patient perspective.

## Supplementary Information

Below is the link to the electronic supplementary material.

Supplementary file1 (DOCX 16 KB)

## Data Availability

No datasets were generated or analysed during the current study.
