# Supplementary material for: Perspectives of Challenges in Counseling for Congenital Heart Defects
Source: Pediatr Cardiol. 2024 Jun 22;46(4):947–55. doi: 10.1007/s00246-024-03520-x (PMC11903537; doi:10.1007/s00246-024-03520-x)
Supplement: Supplementary file 1 — Supplementary file1 (DOCX 16 KB) [file 246_2024_3520_MOESM1_ESM.docx]

**Supplemental Table 1**. Phone Survey

Section 1: Questions about your general prenatal care

1. Did you receive prenatal care, or medical care during your pregnancy?
   1. No
   2. Some (missed several appointments, or prenatal care was started in late second or during third trimester)
   3. Yes
2. Was your first obstetric visit with a nurse midwife, or nurse practitioner, rather than a doctor?
   1. No
   2. Yes
   3. Don’t remember
3. What is the name of the office/clinic/hospital where you received your first prenatal care or ultrasound?
4. What is the ZIP code, or neighborhood, of the office/clinic/hospital where you received your first prenatal care or ultrasound? An example of a neighborhood might include: “Lakeview” or “Hinsdale”

Section 2: Questions about your demographic information

1. How would you identify your race?
   1. White
   2. Black
   3. Asian
   4. American Indian or Alaskan Native
   5. Native Hawaiian or Pacific Islander
   6. More than one race
   7. Other
2. Do you identify as Hispanic?
   1. No
   2. Yes
3. What is the most common language spoken at home?
4. How many years of education, including grade school, have you received? For example, 8 years 8^th^ grade was your last year of school. 12 years means you graduated high school. 16 years means you graduated college. 18 years means you obtained a master’s degree, etc.
5. Were you employed or working in the four weeks prior to delivering your baby?
   1. No
   2. Yes

Section 3: Questions about advanced obstetric care

1. Were you followed by a maternal fetal medicine (MFM, or high-risk obstetrician) specialist before delivering your baby?
   1. No
   2. I saw an MFM specialist for consultation, at least once
   3. Yes, I closely followed with an MFM specialist for the entirety of my pregnancy
2. Did you ever receive a level 2 ultrasound (also called a “20-week” or “anatomic” or “detailed” ultrasound)?
   1. No
   2. Yes
3. Did you ever receive a fetal echocardiogram (an ultrasound that only looks at the heart of the fetus) during your pregnancy?
   1. No
   2. Yes
4. Which of the following made it more difficult for you to obtain a fetal echocardiogram(s)? You can choose multiple options.
   1. I had no issues obtaining my fetal echocardiogram(s)
   2. Hard to remember my appointment time, or no reminders about my appointment
   3. There were not enough appointment time options
   4. Insurance approval and/or payment for fetal echocardiogram
   5. Access to transportation was difficult, and/or the office for the fetal echocardiogram was very far
   6. Difficulty getting time off from work in order to make the appointment
   7. Difficulty getting childcare for my other children in order to make the appointment
   8. I did not feel that the fetal echocardiogram was important
   9. Other (please tell us about other barriers you experienced, in the next question)
5. Is there anything else you’d like to mention about how your congenital heart disease was diagnosed during your pregnancy?
